# Therapeutic Antibodies in Cancer Treatment in the UK

**DOI:** 10.3390/ijms232314589

**Published:** 2022-11-23

**Authors:** Khadiga Eltarhoni, Faddy Kamel, Katrina Ihebunezie, Pasha Nisar, Mikhail Soloviev

**Affiliations:** 1 Department of Biological Sciences, Royal Holloway University of London, Egham, Surrey TW20 0EX, UK; 2 Ashford and St Peter’s Hospitals NHS Foundation Trust, Guildford Road, Chertsey KT16 0RQ, UK

**Keywords:** monoclonal therapeutic antibody, antibody drug conjugates, bispecific antibodies, bispecific T-cell-engager, cancer, cancer treatment

## Abstract

The growing understanding of the molecular mechanisms of carcinogenesis accelerated the development of monoclonal therapeutic antibodies to specifically target multiple cancer pathways. Recombinant protein therapeutics now constitute a large proportion of yearly approved medicines. Oncology, autoimmune diseases and to a smaller degree the prophylaxis of organ transplant rejection are their main application areas. As of the date of this review, 37 monoclonal antibody products are approved for use in cancer treatments in the United Kingdom. Currently, the antibody therapeutics market is dominated by monoclonal immunoglobulins (IgGs). New types of recombinant antibody therapeutics developed more recently include bispecific recombinant antibodies and other recombinantly produced functional proteins. This review focuses on the approved therapeutic antibodies used in cancer treatment in the UK today and describes their antigen targets and molecular mechanisms involved. We provide convenient links to the relevant databases and other relevant resources for all antigens and antibodies mentioned. This review provides a comprehensive summary of the different monoclonal antibodies that are currently in clinical use primarily in malignancy, including their function, which is of importance to those in the medical field and allied specialties.

## 1. Introduction

The 20th century has brought a better understanding of the molecular mechanisms of anti-tumour immunity. The main principle surrounding targeted molecular therapies is based on the understanding that both cancer and stromal cells express many proteins, and other functional ligands that all play a pivotal role in allowing cancer cells to survive and proliferate. Since the development of hybridoma techniques to generate monoclonal antibodies in the 1970s [1], molecular approaches to grafting complementarity determining regions (CDRs) and the concept of humanized antibodies [2], and following the subsequent development of in vitro evolution of antibodies using the phage display technique [3], the interest in immunotherapeutic approaches and in the use of therapeutic antibodies has rocketed. In the last decade, monoclonal therapeutic antibodies became the top-selling molecular therapeutic by value [4]. These molecular therapeutics now constitute a large proportion of yearly approved drugs [5]. Their key application areas cover oncology, autoimmune diseases and in the prophylaxis of organ transplant rejection [6]. A large variety of cancers has been targeted using immunotherapeutic approaches, including leukemia, breast cancer, colon cancer, melanoma, lymphomas, and bladder cancers [7]. In the United Kingdom, 37 monoclonal antibody products are approved for use in cancer treatments. Whilst the antibody therapeutics market is still dominated by monoclonal IgGs, recent developments include the appearance of approved bispecific recombinant antibodies and other recombinantly produced functional proteins. This review provides a comprehensive summary of all the different classes of monoclonal antibodies and their associated uses in the treatment of multiple malignancies. The use of monoclonal antibodies is fast expanding and are showing promising results in some malignancies, especially for malignancies that are resistant to traditional chemotherapy regiments, and are being used in clinical practice for others.

## 2. Therapeutic Antibodies Targeting Antigens Involved in T-Cell Activation, Proliferation, Cytotoxicity and Immune Tolerance

### 2.1. Programmed Cell Death Protein 1 (PD-1)

T-cell membrane proteins that are involved in direct regulation of the T-cell receptors (TCR), such as CD3 complex and other immune-modulatory receptors expressed on the surface of T-cells, represent some of the most exploited cancer-related protein antigens targeted with protein therapeutics, such as monoclonal antibodies. The list also includes other T-cell membrane proteins that are involved in the regulation of TCR function and T-cell activation, that are reviewed further below. Of these, the programmed cell death protein 1 (PD-1), also known as cluster of differentiation protein CD279, is currently the most commonly targeted protein in cancer treatments. There are 12 monoclonal therapeutics targeting PD-1 signalling pathway in use in the USA and Europe, including the UK. PD-1 is a single-pass type I transmembrane protein expressed on the surface of T-cells, which inhibits T-cell activation by binding to the engaged T-cell receptor (TCR) in the immunological synapse. PD-1 contains a single Ig-like motif in its extracellular N terminal topological domain, and one each of the immunoreceptor tyrosine-based inhibitory motif (ITIM) and the immunoreceptor tyrosine-based switch motif (ITSM) in its cytoplasmic topological domain [8]. The normal function of PD-1 protein involves negative regulation of T-cell response, maintaining immune self-tolerance and protection from autoimmunity. PD-1 inhibitory response can be triggered following the binding of the programmed death ligand 1 (PD-L1), also referred to as CD274, and programmed death ligand 1 (PD-L2), also referred to as CD273, expressed by antigen-presenting cells (APC), reviewed below. The PD-1 receptor serves as a key regulator of the immune system and an immune checkpoint to inhibit the immune response by T-cells. Cancer cells may utilize a PD-1-mediated pathway to artificially suppress and escape the T-cell immune response.

**Figure 1:**
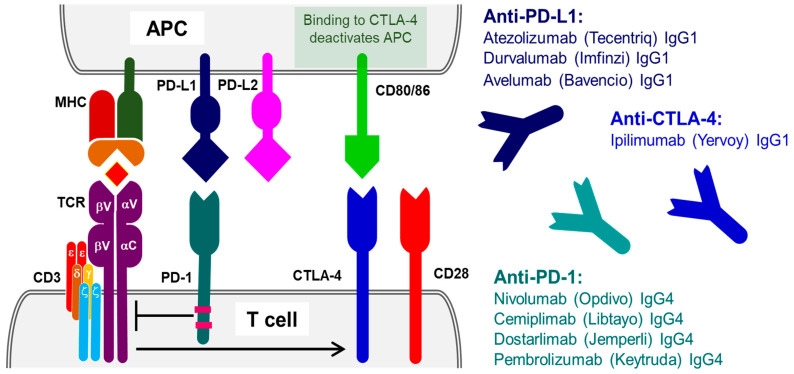
Immune checkpoint inhibitors. Programmed cell death protein 1 (PD-1) expressed on T-cells interacts with programmed death ligands PD-L1 or PD-L2, expressed on the antigen-presenting cells (APC), to downregulate T-cell response, reduce cytokine production by the T-cells, and to block T-cell proliferation. Cytotoxic T-lymphocyte protein 4 (CTLA-4) expresses in response to T-cell activation, following the binding of the receptor complex, made of T-cell receptor (TCR) and CD3, to the antigen presented by the APC MHC. CTLA-4 outcompetes CD28 and binds the T-cell costimulatory ligand CD80/86 and is responsible for negative regulation of T-cell differentiation, proliferation and the immune response.

Given the importance of this checkpoint, the programmed cell death protein 1 (PD-1) attracted the most attention, leading to the development of multiple monoclonal antibodies with nine different approved antibody therapies currently available in the USA and in Europe and a few more are pending approval for clinical use. Of these, four different therapeutic antibodies targeting PD-1 receptor antigen are currently licensed for use in the UK. All four antibodies are unconjugated immunoglobulins targeting PD-1 receptor on the surface of T-cells and aiming to block PD-1 receptor interaction with its ligands PD-L1 and PD-L2. Nivolumab (Opdivo) is a human IgG4 immunoglobulin that was developed by Medarex using their transgenic mice model (‘humanized mouse’). Opdivo is an unconjugated antibody that is currently used in combination to Ipilimumab and chemotherapy or as monotherapy in the treatment of multiple malignancies, including melanoma [9], lung cancer [10,11] and renal cancers [12]. Cemiplimab (Libtayo), is a fully human recombinant monoclonal IgG4 antibody against the PD-1 receptor [13]. Cemiplimab is used in the treatment of various cancers, including cutaneous squamous cell cancer [14], non-small-cell lung cancer [15] and recurrent cervical cancer [16]. Dostarlimab (Jemperli) is another humanized monoclonal IgG4 antibody against the PD-1 receptor, which functions as PD-1 antagonist [17]. Dostarlimab is used primarily in the treatment of endometrial cancer [18]. Pembrolizumab (Keytruda) is another unconjugated humanized IgG4 kappa immunoglobulin that targets PD-1 receptor. That antibody molecule possesses stabilizing sequence alteration in the Fc region, and is used in the treatment of large number of malignancies, including the following: melanoma [19], non-small-cell lung cancer [20], urothelial carcinoma [21], classical Hodgkin lymphoma [22], squamous cell carcinoma of the head and neck [23], colorectal cancer [24], breast cancer [25], endometrial carcinoma [26], cervical cancer [27], biliary tree cancer [28], renal cell cancer [29], esophageal cancer [30], gastric cancer [31], and small intestinal cancer [32] (Figure 1).

### 2.2. Programmed Cell Death Ligand (PD-L1)

The programmed cell death ligands ‘1’ (PD-L1) and ‘2’ (PD-L2), also known as CD274 and CD273, respectively, are expressed by APCs and serve as endogenous protein ligands for the inhibitory receptor PD-1, expressed by T-cells and B-cells [33,34]. Both PD-L1/2 are single-pass type I membrane proteins, with extracellular N termini containing one of each of the Ig-like V-type and C2-type domains, and short intracellular C termini. An alternatively spliced form which may be secreted has been reported for PD-L2 but not for PD-L1. PD-L1/2 binds to the programmed cell death protein 1, which deactivates T-cells. This in turn blocks cytokine production by the T-cells, and blocks T-cell proliferation, reducing apoptosis in regulatory T-cells and overall suppressing the adaptive immune response. Several tumours express high levels of PD-L1/2 which helps the tumour cells evade anti-tumour immunity. These protein ligands have therefore been targeted with the aim of blocking their interaction with PD-1. A number of monoclonal antibodies against PD-L1 have been developed over the last decades.

Three different monoclonal antibodies that target PD-L1 are currently licensed in the UK. Atezolizumab, that is available under the brand name Tecentriq, is an Fc-engineered humanized IgG1. Atezolizumab is an unconjugated antibody, currently used in the treatment of various cancers. Treatment with atezolizumab is still being trialled in patients with urothelial carcinoma and is yet to be proven to show longer term survival rates but is still in phase III trials [35]. In non-small-cell lung carcinoma, combination atezolizumab treatment has been shown to improve overall survival rates in patients with EGFR mutations [36]. In the treatment of advanced triple-negative breast carcinoma, combination atezolizumab treatment has been shown to increase disease-free survival by over 4 months [37]. In those patients with unresectable hepatocellular carcinoma, combination atezolizumab treatment results in longer progression-free survival of patients [38].

Durvalumab, that is available under the brand name Imfinzi, is another unconjugated human IgG1 kappa immunoglobulin against PD-L1 which is currently licensed in the UK for the treatment of non-resectable non-small-cell lung cancer (NSCLC) [39]. In the treatment of this cancer, it is shown to significantly improve progression-free survival in patients with stage III disease [40]. There are current trials in the use of Durvalumab in the treatment of patients with other cancers including hepatocellular carcinoma [41], biliary tree carcinomas [42], head and neck squamous cell carcinomas [43] and cervical carcinoma [44]. Another anti PD-L1 monoclonal antibody is Avelumab, which is available under the brand name Bavencio. Avelumab is a fully human unconjugated IgG1 against PD-L1 that is currently used in the treatment of urothelial carcinoma [45,46], renal cell carcinoma [47,48] and Merkel cell carcinoma [49,50] as monotherapy (summarized in Figure 1).

### 2.3. Cytotoxic T-Lymphocyte Protein 4 (CTLA-4)

Cytotoxic T-lymphocyte protein 4 (CTLA-4), also known as Cytotoxic T-lymphocyte-associated antigen 4 CD152, and previously as CELIAC3 or IDDM12, is a tightly regulated single-pass type I membrane protein expressed in T-cells and responsible for negative regulation of T-cell differentiation, proliferation and of the immune response [51]. CTLA-4 function depends on regulatory T-cells, where CTLA-4 is expressed constitutively, and it may also exert its downregulatory function onto the activated T-cells directly independent of the regulatory T-cells (reviewed in [52]). CTLA-4 serves as high-affinity antigen-independent receptor for the T-cell costimulatory ligand CD80/86, capable to outcompete the antigen-dependent binding of CD28—the other protein-ligand of CD80/86. Upon activation of the TCR, CTLA-4 is transported to the surface of T-cells where it binds to CD80/86. That binding deactivates the APC and downmodulates the T-cell response. Normal CTLA-4 function may therefore cause inhibition of antitumor T-cells, and such a mechanism is hijacked by tumours allowing the evasion of anti-tumour immunity and thus facilitating tumour survival.

A monoclonal immunotherapy targeting CTLA-4 protein (Ipilimumab) was the first-in-class checkpoint inhibitor to receive FDA approval. Ipilimumab is a fully human anti-CTLA-4 monoclonal IgG1 kappa immunoglobulin, which binds to and blocks the CTLA-4 receptors on the T-cells (Figure 1). Transient blockade of the CTLA-4 allows for T-cells to proliferate and be activated at a lower threshold than the physiological status. Ipilimumab, available under the brand name Yervoy, remains the only antibody targeting CTLA-4 which is licensed in the UK for the treatment, either as a monotherapy or in combination with other immunotherapeutic or cytotoxic chemotherapy, of melanoma where five-year survival rates can be seen in up to 52% of patients with advanced disease [9]. In advanced renal cell carcinoma, there is shown to be an increase in 18-month survival rates from 60% to 75% with the combined use of ipilimumab [12]. In colorectal carcinoma ipilimumab is being trialled in the use of advanced metastatic disease showing mismatched repair and microsatellite instability deficiencies [53,54]. Ipilimumab is being trialled as first-line treatment in non-small-cell lung cancer and is being shown to produce longer overall survival rates compared to conventional treatments [10,11]. In the treatment of non-resectable malignant pleural mesothelioma, ipilimumab has been shown to provide long-term overall survival benefit [55].

## 3. Antibody Therapeutics Targeting B-Cell and T-Cell Antigens

### 3.1. B-Lymphocyte Surface Antigen B4 (CD19)

CD19, also known as B-lymphocyte surface antigen B4, T-cell surface antigen Leu-12 and Differentiation antigen CD19, is a transmembrane protein containing two IG-like domains and expressed in B-cells that acts as a coreceptor for the B-cell antigen receptor complex (BCR) on B-lymphocytes [56]. CD19 is involved in B-cell antigen receptor-mediated signal transduction, regulation of B-cell activation, proliferation, and Ig-mediated immune response. CD19 is expressed in the lineage of B-cell differentiation from haemopoietic cells, and its expression is maintained during neoplastic transformation.

**Figure 2:**
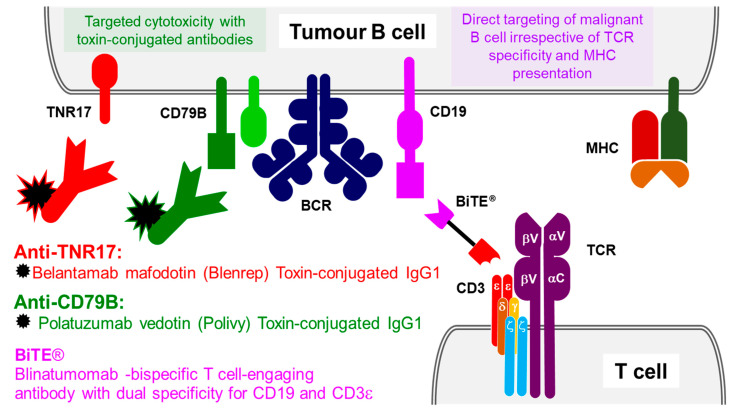
Antibody therapeutics targeting B-cell and T-cell antigens. Targeting tumour B-cells with monoclonal antibodies against tumour necrosis factor receptor superfamily member 17 (TNR17) conjugated to anti-mitotic maleimidocaproyl monomethyl auristatin F (mcMMAF) (coloured red). Targeting tumour B-cells with monoclonal antibodies against B-cell antigen CD79B of the BCR complex, conjugated to anti-mitotic monomethyl auristatin E (MMAE) (coloured green). Bispecific T-cell-engaging antibodies (BiTE) has dual specificity for CD19 and CD3 and redirects polyclonal cytotoxic T lymphocytes toward the tumour.

Tafasitamab is a humanized Fc-modified cytolytic IgG that is available under the brand name Monjuvi. Tafasitamab binds to the CD19 surface antigen, initiating the lysis of B lymphocytes by immune-mediated response and apoptosis. Currently, it is only licensed to be used as monotherapy for the treatment of diffuse large B-cell lymphoma. It has been suggested that the addition of lenalidomide (an immunomodulatory agent that augments bone marrow natural defences against cancer cells) as a treatment in refractory cases of diffuse large B-cell lymphoma can have a beneficial effect, however, this has only undergone a phase II trial and is still awaiting phase III studies [57]. Loncastuximab tesirine is a humanized monoclonal antibody against CD19 that targets B-cells [58], which is stochastically conjugated via cleavable maleimide linker to an anticancer cytotoxin pyrrolobenzodiazepine dimer (PBD). Loncastuximab tesirine is available under the brand name Zynlonta. As the main function of this antibody is to target B-cells, it is used primarily in the treatment of large B-cell lymphoma [59] (Figure 2).

### 3.2. CD3 Co-Receptor

CD3 is a protein complex made of CD3 gamma, delta, zeta and epsilon subunits (also known as T-cell receptor gamma, delta, zeta chains and T-cell surface antigen T3/Leu-4 epsilon chain, respectively) in a complex with T-cell receptor (TCR) alpha and beta chains [60,61,62,63]. The assembled CD3 co-receptor contains a few extracellular Ig-like motifs and a few more intracellular immunoreceptor tyrosine-based activation motifs (ITAM) and is involved in the activation of T helper and cytotoxic T-cells. Blinatumomab, (Blincyto), which was approved by NICE in 2019 [64], is used for the treatment of acute lymphoblastic leukaemia (ALL). Blinatumomab is an engineered bispecific T-cell engager (BiTE) protein made of two immunoglobulin domains connected with a short protein linker. One of these two domains is a single-chain variable antibody variable domain (ScFv) against CD19 B-lymphocyte surface antigen (expressed on B-cells), connected to another ScFv against CD3 co-receptor complex on T-cells. Therefore, such a bispecific T-cell engager molecule (Blinatumomab BiTE) targets two antigens (CD19 and CD3) simultaneously. Such an entirely artificial bispecific engager activates CD3-positive cytotoxic T-cells and also brings them directly to the malignant immature lymphoblasts, proliferated in the ALL. Blinatumomab BiTE will also target resting B-cells expressing the CD19 antigen, ultimately leading to the lysis and elimination of all CD19+ cells. The ingenuine design of the Blinatumomab allows it to bypass the native mechanism utilized by the adaptive immune system, that relies on the specific TCRs, whilst achieving the same outcome—activation of T-cells and their targeting to CD19+ lymphoblasts. Blinatumomab is one of the only two bispecific therapeutic antibodies approved for use in the UK and the only ScFv-based BiTE construct out of a handful of bispecific antibodies approved for use in Europe and USA.

### 3.3. B-Lymphocyte Antigen CD20

B-lymphocyte antigen CD20, is also known as B-lymphocyte surface antigen B1, Bp35, Leukocyte surface antigen Leu-16, and a membrane-spanning 4-domains subfamily A member 1. CD20 is a multi-pass transmembrane non-glycosylated phosphoprotein, constitutively associated with lipid rafts in B-cell membranes [65]. CD20 plays role in development, differentiation, and activation of B-lymphocytes. Membrane-associated CD20 is involved in regulating calcium influx and apoptosis. The first CD20 approved monoclonal antibody is Rituximab which targets CD20. Rituximab (MabThera, Rixathon, Truxima) is human/Murine, chimeric monoclonal IgG1 initially used exclusively in the treatment of non-Hodgkin B-cell lymphoma by binding to CD20 antigen which is upregulated in malignant B-cells. The binding then initiates the process of elimination of CD20-positive cells, either by apoptosis, activation of the complement cascade or by triggering cell-mediated immune response, activation of the complement cascade [66]. Rituximab is used in combination with other chemotherapy regimens including cyclophosphamide, doxorubicin, vincristine, and prednisone (CHOP) commonly known as the R-CHOP regimen. However, it is now used for other autoimmune disorders such as rheumatoid arthritis and Pemphigus vulgaris [67,68]. Given the high specificity of CD20 and the success Rituximab had, a number of other monoclonal antibodies were developed and are currently used in the UK in the treatment of B-cell lymphomas and leukaemia that are refractory to the first-line treatment with rituximab or in patients were first line treatment is not possible.

**Figure 3:**
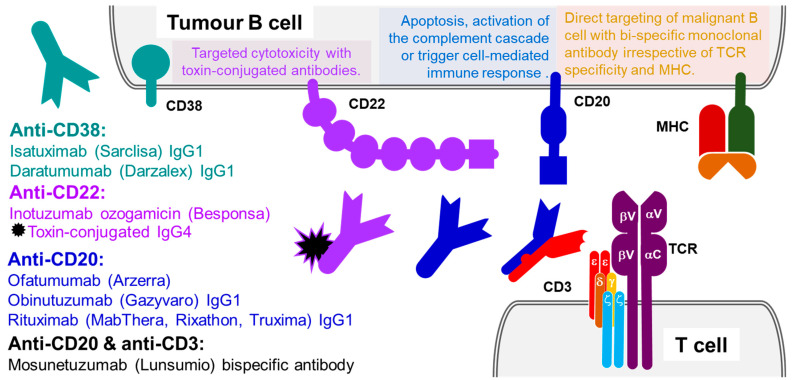
Antibody therapeutics targeting B-cell and T-cell antigens. Targeting tumour B-cells with unconjugated monoclonal antibodies against ADP-ribosyl cyclase/cyclic ADP-ribose hydrolase 1 (protein CD38, coloured teal) or unconjugated monoclonal antibodies against B-lymphocyte antigen CD20 (coloured blue). Targeting tumour B-cells with monoclonal antibodies against B-cell receptor CD22 conjugated to a cytotoxic antibiotic N-acetyl-gamma-calicheamicin dimethylhydrazide (coloured purple). Bispecific antibody simultaneously targets B-lymphocyte antigen CD20 and CD3 epsilon subunit of the CD3-TCR complex on T-cells (coloured in blue-red), and redirects polyclonal cytotoxic T lymphocytes toward the tumour.

Obinutuzumab that is available under the brand name Gazyvaro, is a third-generation glycoengineered unconjugated humanized type II IgG1 immunoglobulin against MS4A1 (CD20) protein antigen, derived from parental mouse antibody (B-Ly1) [69]. It is used in the treatment of three haematological malignancies including follicular lymphoma [70], chronic lymphocytic leukaemia [71] and in refractory diffuse B-cell lymphoma [72]. Ofatumumab, that is available under the brand name Arzerra, is an anti-CD20 monoclonal unconjugated antibody that induces antibody-dependent and complement-mediated cytotoxicity in CD20-expressing B lymphocytes [73]. It is used in the treatment of chronic lymphoid leukaemia [74]. Mosunetuzumab is a CD20 and CD3 bispecific monoclonal antibody that initiates B-cell elimination by bringing the targeted B-cells towards and binding them directly to T-cells. Mosunetuzumab has been approved by the European Medicines Agency for the treatment of adult patients with relapsed or refractory (R/R) follicular lymphoma (FL), who have received at least two prior systemic therapies. Mosunetuzumab is currently under investigation in the UK to evaluate its use in refractory cases of follicular lymphomas. Recommendations for Mosunetuzumab UK use are expected from NICE in due course. Elsewhere, Mosunetuzumab is available under the brand name Lunsumio as a concentrate, used as a solution for intravenous infusion (Figure 3).

### 3.4. B-Cell Receptor CD22

A B-cell receptor CD22 is also known as B-lymphocyte cell adhesion molecule (BL-CAM), Sialic acid-binding Ig-like lectin 2 (Siglec-2) and T-cell surface antigen Leu-14. CD22 protein contains multiple extracellular Ig-like domains and four cytoplasmic immunoreceptor tyrosine-based inhibitory motifs (ITIM) [75]. The latter are involved in negative regulation of the immune response. The extracellular Ig-like sialic acid binding lectins bind sialylated glycoproteins and are involved in the regulation of B-cell antigen receptor signalling, B-cell to B-cell interactions and activation of B-cells. Inotuzumab ozogamicin, available under the brand name Besponsa, is a humanised IgG4 kappa monoclonal antibody raised against CD22 protein antigen that is covalently linked to a cytotoxic antibiotic N-acetyl-gamma-calicheamicin dimethylhydrazide. Inotuzumab ozogamicin ADC is used to treat different types of haematological malignancies by targeting CD22 receptor-expressing cells with the cytotoxic agent directly. This ADC does not target normal cells that do not express CD22. Inotuzumab ozogamicin is currently used for acute lymphoblastic leukaemia to target CD22-positive precursors and as a monotherapy for relapsed or refractory leukaemia [76].

### 3.5. ADP-Ribosyl Cyclase CD38

ADP-ribosyl cyclase/cyclic ADP-ribose hydrolase 1 protein CD38, also known as 2′-phospho-ADP-ribosyl cyclase/2′-phospho-cyclic-ADP-ribose transferase, ADP-ribosyl cyclase 1 (ADPRC 1), cADPr hydrolase 1 and T10, belongs to ADP-ribosyl cyclase family. CD38 is a single pass type II transmembrane protein expressed on the surface of immune cells and is involved in many functions including B-cell receptor signalling, regulation of B-cell proliferation, regulation of cell growth, apoptosis, Ca signalling, and multiple other signalling events [77]. CD38 protein has only a very small cytoplasmic N terminal topological domain and a large glycosylated extracellular C-terminal topological domain, heavily crosslinked with disulphide bonds. CD38 protein is expressed in range of tissues as well as in malignant lymphoma and neuroblastoma and is abundant on B-cells and T-cells during early and late stages of their maturation. Isatuximab, available under the brand name Sarclisa, is an unconjugated IgG1 monoclonal antibody that binds to the glycoprotein CD38, expressed on the surface of haematopoietic and tumour cells [78]. It is used in combination with either anti-angiogenic drugs or with selective proteasome inhibitors, in the treatment of multiple myeloma [79]. Isatuximab, is always used in combination with glucocorticoid dexamethasone. Another anti-CD38 therapeutic is Daratumumab, which is available in the UK under the brand name Darzalex. Daratumumab is also an unconjugated human monoclonal IgG1 (kappa) antibody that binds to the glycoprotein CD38. Daratumumab may be administered by subcutaneous injections or by intravenous administration, two different formulations and therefore available. Daratumumab is used in the treatment of multiple myeloma [80] in newly diagnosed patients or in patients with multiple myeloma who have received prior therapies.

### 3.6. B-Cell Antigen CD79B

B-cell antigen receptor complex-associated protein CD79B, also known as B-cell-specific glycoprotein B29, Ig-beta and immunoglobulin-associated B29 protein, is a single-pass type I transmembrane protein receptor expressed in a number of lymphomas. CD79B is part of a B-cell antigen receptor complex, where a heterodimer made of disulphide-linked CD79 alpha and beta subunits are associated with antigen-specific surface immunoglobulin on the B-cell surface [81]. CD79B possesses one extracellular Ig-like V-type domain and one cytoplasmic ITIM motif. CD79B contributes to the adaptive immune response and B-cell differentiation by mediating signal transduction cascade activated by the B-cell antigen receptor complex (BCR), leading eventually to antigen presentation.

Polatuzumab vedotin is a humanized monoclonal IgG1 antibody covalently conjugated to an anti-mitotic agent monomethyl auristatin E (MMAE). The cytotoxic agent is released inside the cells following internalization of the receptor-ligand complex. Polatuzumab is currently undergoing trials to be used as combination therapy alongside Rituximab (therapeutic antibody) and with other low molecular weight chemotherapy medications such as either Bendamustine or Doxorubicin (the latter in combination with immunosupressants Cyclophosphamide and Prednisone) in treatment of diffused B-Cell Lymphoma [82].

### 3.7. Tumour Necrosis Factor Receptor Superfamily Member 17 (TNR17)

Tumour necrosis factor receptor superfamily member 17 (TNR17), also known as B-cell maturation antigen (BCMA) and CD269, is a single-pass type III membrane protein, expressed in mature B lymphocytes, but not in T-cells or monocytes [83]. TNR17 is a receptor for B-cell activating factor (BAFF), also known as B Lymphocyte Stimulator (BLyS), TNF- and APOL-related leukocyte expressed ligand (TALL-1) and or CD257, which belongs to the TNF receptor superfamily and is important for B-cell development, autoimmune response and is involved in the regulation of humoral immunity. TNR17 has been linked to multiple myeloma, acute monocytic leukaemia, acute myelomonocytic leukaemia (AMML), T-cell lymphoma, follicular lymphoma and autoimmune disorders, and it is used as a prognostic marker as its overexpression is linked to poorer outcomes [84]. Belantamab mafodotin (Blenrep) is an afucosylated humanized monoclonal IgG1 immunoglobulin kappa generated against B-cell maturation antigen TNR17, conjugated to maleimidocaproyl monomethyl auristatin F (mcMMAF), which is a cytotoxin and is administered by infusion. Binding of Belantamab to the targeted cell results in cell lysis. Belantamab is a third-line drug used for treating multiple myeloma in the UK.

## 4. Antibody Therapies against Other Blood Cell Antigens and Vasculature

### 4.1. Lymphocyte Activation Antigen CD30

**Figure 4:**
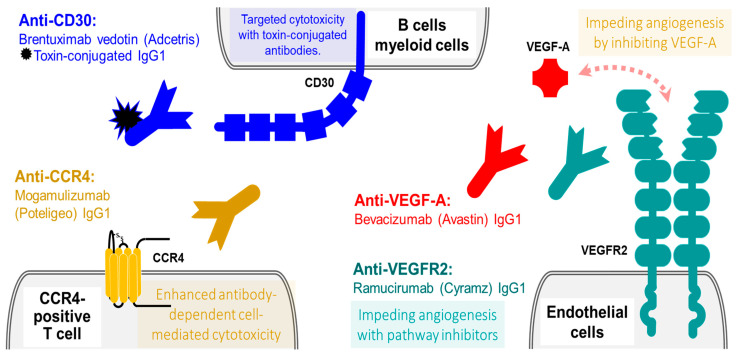
Antibody therapies against other blood cell antigens and vasculature. Targeting CD38 with cytotoxic drug-conjugated antibodies (**top left**). Targeting CCR4 with monoclonal unconjugated antibodies (**bottom left**). Targeting VEGF pathways with monoclonal unconjugated antibodies against VEGF-A growth factor and with monoclonal unconjugated antibodies against VEGFR2 receptor (**right**).

Tumour necrosis factor receptor superfamily member 8, also known as CD30L receptor, Ki-1 antigen, or Lymphocyte activation antigen CD30, is a single-pass type I membrane protein member of the tumour necrosis receptor family [85]. An alternatively spliced isoform of CD30 exists as a soluble cytoplasmic protein. The extracellular topological domain of the long CD30 proteins contains multiple cysteine-rich repeats. Following binding to its ligand, CD30 assembles in trimers, leading to the induction of NF-κB-dependent downstream signalling, that affects a variety of cellular processes, including cell proliferation, survival, as well as apoptosis. The expression of CD30 depends on the transcription factor JunB but could be also upregulated by mitogens or viral stimulation. CD30 is specifically and abundantly expressed by immune cells especially Reed-Sternberg cells (Anaplastic large-cells), typical for Hodgkin’s lymphoma, and in embryonal carcinoma, which makes CD30 an ideal target for anticancer monoclonal antibody therapy (Figure 4).

Brentuximab vedotin (Adcetris) is a recombinant chimeric IgG1 monoclonal antibody, targeting the long isoform of the membrane-associated CD30, conjugated to three to five units of the monomethyl auristatin E (MMAE). MMAE is a potent antimitotic agent that functions via disturbing the microtubules and enhancing apoptosis [86]. As this receptor is specifically expressed by hematologic cells the drug targeting it is used in a specific hematologic malignancy which is Hodgkin’s lymphoma and Anaplastic large-cell lymphoma [87]. Binding of Brentuximab vedotin to CD30-expressing cells results in the internalization of the antibody and release of the bond cytotoxic MMAE into the cell cytosol, leading to the apoptosis of the tumour cell [88].

### 4.2. Mogamulizumab (Poteligeo): A Humanized IgG1 Therapeutic Antibodies against C-C Chemokine Receptor Type 4 (CCR4)

C-C chemokine receptor type 4 (CCR4), which is also known as CD194, is a G protein-coupled receptor expressed mainly in the thymus, peripheral blood leukocytes, T-cells, basophils, and platelets and to a lower degree in a range of other cells and tissues [89]. CCR4 is moderately specific for the skin effector T-cells that are also known as skin homing memory T-cell. These cells co-express Cutaneous lymphocytes antigen (CLA) along with CCR4 both of which binds to ligands such as E-selectin on the dermal venules, CC-chemokine ligand 17 and CCL22 [90]. These interactions are essential in allowing the T-cells and to enter in the dermis and epidermis layers. These skin-homing memory T-cells mediate the inflammatory response to injury in the skin and the dysregulation in the function of these cells is believed to be the key to developing number of skin diseases including cutaneous T-cell Lymphomas (CTCLs). CTCLs are a heterogenous group of proliferative disorders that is characterised by the presence of malignant T-cells the most common types of CTCLs (Mycosis fungoides and Sézary syndrome) are shown to express increased levels of CCR4 [90]. The fact that it is relatively specific for skin homing T-cells and over expressed in certain types of CTCLs have made it an ideal target for monoclonal antibodies [91]. In spite of it being a promising target, only one anti-chemokine receptor CCR4 that has been approved to be used against this disease and that being the drug Mogamulizumab (Poteligeo) is a humanized unconjugated afucosylated monoclonal IgG1 antibody with enhanced antibody-dependent cell-mediated cytotoxicity, that is administered intravenously for selected patients with certain types of cutaneous T-cell lymphoma [92]. Mogamulizumab has, in selected cases, significantly improved quality of life by reducing symptom burden such as itching, however, the evidence that has been evaluated thus far shows that Mogamulizumab does not improve overall survival [93].

### 4.3. VEGF Pathway Inhibitors: VEGF and VEGFR

Angiogenesis is an essential process in cancer growth, invasion and migration, the process is driven by number of receptors and ligands, the most important of which are vascular endothelial growth factors (VEGF) and their receptors (VEGFR). VEGFA is a small protein growth factor involved in regulation of angiogenesis, vasculogenesis and endothelial cell growth, proliferation, and migration [94]. VEGFA signalling inhibits apoptosis and increases vascular permeability. Multiple isoforms of VEGFs exist. VEGF expression may be induced by hypoxia, whilst under normal conditions it is regulated by multiple factors. VEGFA binds to VEGFAR-1 and VEGFAR-2 receptors [95,96]. Some of the VEGF isoforms are involved in motor neuron axon guidance through their interactions with NRP1 receptor.

Vascular endothelial growth factor receptor 2 (VEGFR-2) is also known as foetal liver kinase 1 (FLK-1), Kinase insert domain receptor (KDR), protein-tyrosine kinase receptor flk-1, and CD309. Multiple isoforms of VEGFR-2 exist, including a cell surface single-pass type I membrane protein as well as secreted variants [96]. VEGFR-2 is a receptor for VEGFA, VEGFC and VEGFD factors [94,97,98] which also acts as a tyrosine-protein kinase, involved in embryonic haematopoiesis and tumour angiogenesis. VEGFR-2 dimerizes following the binding of its ligands, causing mitogenic and survival signalling events, affecting endothelial cell survival, proliferation, and migration. VEGFR-2 is overexpressed in neovascular tumour endothelial cells in number of cancers. Low VEGF expression and reduced VEGFR signalling induces apoptosis in the cells. Therefore, number of strategies have been developed to antagonize the effect of VEGF, one of these is Bevacizumab (Avastin) which is used in the UK treatment different cancers [99]. Bevacizumab is an unconjugated humanized monoclonal IgG1 immunoglobulin that blocks angiogenesis by inhibiting vascular endothelial growth factor A (VEGF-A). Bevacizumab is used to treat colorectal cancers, non-squamous non-small-cell lung cancer, primary peritoneal cancer, renal cell carcinoma [100], epithelial ovarian cancers [101], cervical carcinoma [102], metastatic breast cancer [103] and hepatocellular carcinoma [104]. Another drug that aims to inhibit the VEGF lead angiogenesis is Ramucirumab (Cyramz), which is fully human monoclonal IgG1 immunoglobulin raised against VEGFR2. In the UK it is used in the treatment of oesophageal and gastric cancer, metastatic colorectal cancer, non-small-cell lung cancer. All systemically administered VEGF pathway inhibitors increase the risk of arterial wall abnormalities including aneurysm and dissection. Mortalities have been reported due aortic aneurysm and arterial dissection therefore clinical are advised to consider patients carefully with the aim to reduce other modifiable risk factors such as smoking and optimizing blood pressure. Furthermore, it is advised to avoid elective surgeries while on treatment, and to discontinue the administration for at least 4 weeks prior to the surgery and to delay the start of the treatment until the wound has healed. As anti-VEGF treatment is reported to cause delayed wound healing [105].

## 5. Ubiquitously Expressed Cancer Targets for Anticancer Antibody Therapies

### 5.1. Therapeutic Antibodies against Epidermal Growth Factor Receptor Family (EGFR)

**Figure 5:**
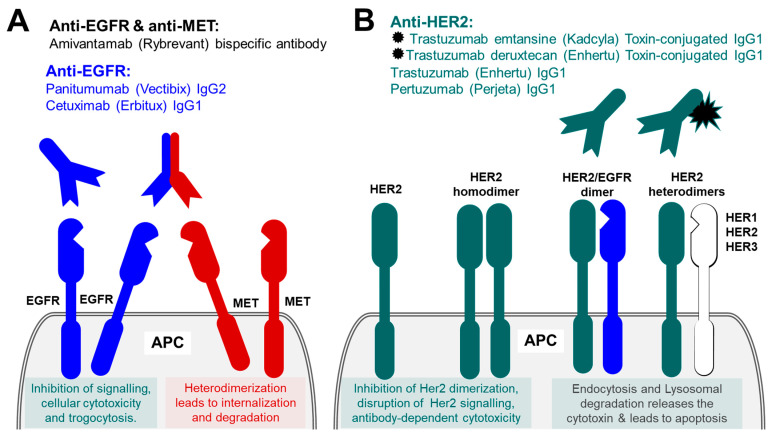
Ubiquitously expressed cancer targets for anticancer antibody therapies. (**A**) Targeting EGFR with monoclonal unconjugated antibodies and bispecific anti-EGFR and anti-MET antibodies. (**B**) Targeting HER2 with monoclonal unconjugated antibodies and cytotoxic drug-conjugated antibodies.

The epidermal growth factor receptor (EGFR), also known as proto-oncogene c-ErbB-1, receptor tyrosine-protein kinase erbB-1 (Her1 in humans), represents another common target for anti-cancer immunotherapeutic. Epidermal growth factor receptor (EGFR) is a member of the report tyrosine kinase (RTK) family of receptors, which includes structurally related ErbB1 (Her1), ErbB2 (Her2), ErbB3 (Her3) and ErbB4 (Her4). These also represent the first group of RTK that was identified and was linked to cancer [106]. EGFR is a single-pass type I transmembrane protein with a large hydrophilic extracellular glycosylated portion that serve as ligand biding site, and intracellular topological domain with a juxtamembrane domain responsible for dimerization, phosphorylation and activation of EGFR, and a protein kinase domain. EGFRs are heavily involved in the regulation and activation of diverse and essential cellular processes including cell survival, proliferation, differentiation, migration, and apoptosis. EGFR is activated following binding of epidermal growth factor or transforming growth factor α (TGFα). ErbB2/Her2 has no known ligand and is believed to be expressed constitutively and activated due to homo- or hetero-dimerization [107]. EGFR somatic mutations are linked to several cancers. These were first identified in the lung tissue and were later discovered in many other cancers, including ovarian, colorectal, renal, head and neck, prostate, and biliary tree malignancies [108]. Overall, 90% of EGFR mutation are represented by a point deletion in exon 19 and a point mutation L858R in exon 21 [109]. Other less common mutation shave also been identified including exon 20 insertions mutation, exon 18-point mutations among others [110]. ErbB3/Her3 expression appear to be linked to about 50–70% of lung, colorectal and breast carcinomas. About 30% of primary breast carcinomas express ErbB2/Her2 receptor, and about 22% of colorectal cancers express ErbB4/Her4 receptors [111,112]. Over the last few decades EGFR has been heavily investigated as a potential target for anticancer immunotherapy. High EGFR expression in the most common cancers, made EGFR family a tenable target for a number of monoclonal antibodies, aiming to block EGFR binding sites on the extracellular domain of the receptor or to prevent dimerization leading to the inhibition of intracellular tyrosine kinase activity and preventing the growth of EGFR-expressing tumours (Figure 5A).

Cetuximab (Erbitux) is a chimeric (mouse/human) unconjugated IgG1 antibody that was first approved by FDA in 2004. Cetuximab is used for colorectal cancer and head and neck squamous cell carcinoma [113]. It is approved for use as a monotherapy or in combination with other agents for example chemotherapy or radiotherapy [114]. Panitumumab (Vectibix) is another monoclonal antibody that targets EGFR. Panitumumab is a fully human unconjugated IgG2 generated using Abgenix’s XenoMouse platform technology. Currently, it is only recommended for use in colorectal cancer as a monotherapy or combined with other agents. Both cetuximab and panitumumab are thought to be less toxic to the body systems, however, they are associated with a long list of side-effects that are related to their effect on the EGFR on healthy tissues, these include very common but less serious reactions such as alopecia, constipation, dry mucous membranes, to uncommon but very serious reaction such as angioedema, severe cutaneous reactions and anaphylactic reaction [115,116]. All EGFR targeting drugs has an attached warning of keratitis and ulcerative keratitis which if not managed promptly could result in corneal perforation and subsequent blindness if not treated promptly.

Amivantamab, available under the brand name Rybrevant, is a human IgG1-based bispecific monoclonal antibody which simultaneously targets both EGFR and the mesenchymal–epithelial transition (MET) receptor expressed on the surface of epithelial cells in non-small-cell lung cancer (NSCLC). MET receptor, which is also known as hepatocyte growth factor receptor (HGF receptor), scatter factor receptor (SF receptor), tyrosine-protein kinase Met, and proto-oncogene c-Met, is a receptor tyrosine kinase and a proto-oncogene. MET receptor is a single-pass type I membrane protein expressed on the surface of NSCLC cells [117]. MET receptor binds its ligand hepatocyte growth factor [118], also known as Hepatopoietin-A, and scatter factor (SF), and is involved in the regulation of cell proliferation, morphogenesis and survival. Amivantamab is used as first treatment for adults with EGFR exon 20 insertion NSCLC as well as other EGFR mutations in NSCLC following development of resistance to EGFR tyrosine kinase inhibitors. Amivantamab induces immune-mediated activity through multiple mechanisms. The binding of Amivantamab to their antigens reduces their respective ligand binding and therefore signalling by these receptors. Due to it bispecific nature, the antibody prevents homodimerization of EGFR and MET receptors (necessary for their oncogenic signalling), by forcing heterodimerization of these proteins, their internalization and subsequent degradation. Amivantamab also stimulates antibody Fc-dependent cellular cytotoxicity mediated by natural killer cells and trogocytosis by Macrophages.

### 5.2. Therapeutic Antibodies against ErbB2/Her2

Receptor tyrosine-protein kinase erbB-2, also known as Proto-oncogene c-ErbB-2, Proto-oncogene Neu, Metastatic lymph node gene 19 protein (MLN 19), human epidermal growth factor receptor 2 and CD340, is a single-pass type I membrane protein member of the ErbB family of plasma membrane-bound receptor tyrosine kinases (isoform 1) [119]. Homo- or hetero-dimerization of ErbB2 leads to its activation, autophosphorylation and activation of a number of signalling pathways, ultimately leading to cell growth and cell proliferation (Figure 5B). Two alternatively spliced isoforms of this protein are missing the transmembrane region and are confined to the cell cytoplasm/nucleus, there also exists a secreted isoform. Nucleus-targeted isoforms are involved in transactional activation of the cyclin-dependent kinase inhibitor 1 (CDKN1A) and of rRNAs, and stimulate protein synthesis and cell growth.

Trastuzumab (Enhertu) is humanized IgG1 unconjugated antibody, that binds to the extracellular domain IV of a cell membrane-bound glycoprotein Her2, over-represented in 20–30% of breast cancers (Her2-positive cells), which prevents Her2 homo- and hetero-dimerization, required for cell proliferation, angiogenesis, and motility. Trastuzumab has been recommended and widely used for selected group of breast cancer patients who have amplification of ErbB2/Her2 (Her2-positive). Trastuzumab is marketed under many trade names such as Herceptin, Herzuma and Ontruzant. Originally it was used only for treating Her-2 positive breast cancers, but later the range of applications was expanded to include ErbB2/Her2-positive gastro-oesophageal cancers. The binding of Trastuzumab to ErbB2/Her2 protein antigens on the target cell surface inhibit ErbB2/Her2 receptor signalling and led to antibody-dependent cytotoxicity. Another Humanized IgG1 that targets ErbB2/Her2 is Pertuzumab (Perjeta), which in the UK is licensed for Her2-positive breast cancer to be used in combination with trastuzumab and chemotherapy for neoadjuvant treatment of locally advanced breast cancer or inflammatory breast cancer or early breast cancer with high recurrence rate. Pertuzumab binds to the Her3 binding domain of Her2 and acts as inhibitor of Her dimerization, thus disrupting Her-mediated signalling in HER2-positive breast cancers. The combination of these antibodies with chemotherapy improves survival, increases the length of disease-free period and reduces the risk of disease recurrence [120,121,122]. The same Trastuzumab antibody is also used in antibody drug conjugate (ADC) formats. Currently in the UK there are two formats of (ADC) used for breast cancer—Trastuzumab emtansine and Trastuzumab deruxtecan. Trastuzumab emtansine, previously known as Trastuzumab-DM1 (T-DM1), and currently available under the brand name Kadcyla, is the IgG1 monoclonal Trastuzumab covalently linked to a cytotoxic agent Mertansine (a tubulin inhibitor), also known as Maytansinoid DM1, or just DM1. Trastuzumab emtansine binds to ErbB2/Her2 protein antigens on the target cell surface and stimulates receptor-mediated endocytosis and Lysosomal degradation, which results in the release of the cytotoxic Mertansine. The subsequent inhibition of microtubule function eventually leads to the apoptosis. Trastuzumab emtansine also acts in a manner similar to unconjugated Trastuzumab, by inhibiting the receptor signalling and stimulating antibody-dependent cytotoxicity. Trastuzumab emtansine is indicated for use in ErbB2/Her2-positive, early stage and metastatic breast cancers. Trastuzumab deruxtecan, currently available under the brand name Enhertu, is the IgG1 monoclonal Trastuzumab linked through a cleavable peptide linker to a few molecules of the anti-neoplastic topoisomerase I inhibitor deruxtecan (DXd, a derivative of exatecan). Trastuzumab deruxtecan is indicated for use in patients with unresectable or metastatic HER2-positive breast cancer. ErbB2 protein is overexpressed in many cancers and have been exploited as a convenient tumour marker and target in many anti-cancer immunotherapies and the number of anti ErbB2/Her2 therapies continues to grow. One other such chimeric unconjugated IgG1 monoclonal antibody is approved for use by FDA for use with ErbB2/Her2-positive metastatic breast cancer is Margetuximab (Margenza). That treatment has not been approved to use in the UK or EU. One other anti-ErbB2/Her2 antibody Trastuzumab duocarmazine is awaiting FDA approval in the early 2023.

## 6. Other Cancer Targets

### 6.1. Trophoblast Antigen 2 (TROP2)

**Figure 6:**
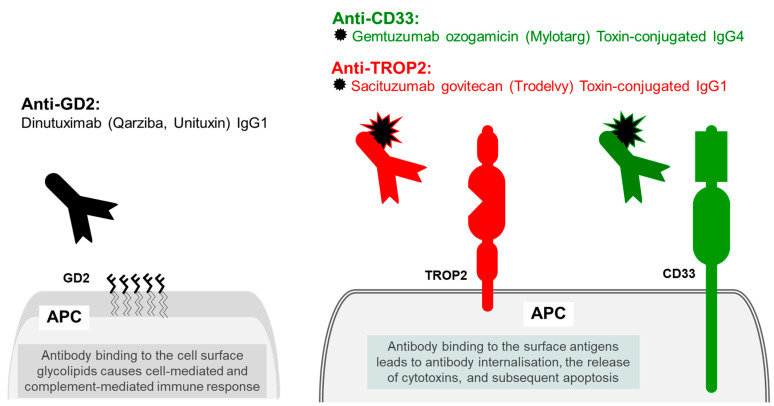
Other cancer targets. Epithelial antigen TROP2 is targeted with monoclonal antibody conjugated to topoisomerase inhibitor SN-38. Myeloid cell surface antigen CD33 is targeted with monoclonal antibody conjugated to a cytotoxic antibiotic N-acetyl gamma calicheamicin. Unconjugated monoclonal antibody targeting of tumour-specific disialoganglioside.

Trophoblast antigen 2 (TROP2) is also known as epithelial glycoprotein 1 (EGP1) tumour-associated calcium signal transducer 2 (tacstd2), and membrane component 1 surface marker 1 (M1S1). TROP2 antigen is involved in large numbers of cell functions. During development, TROP2 expression is essential in development of the internal organs, including the lungs, kidneys, and the gastrointestinal tracts. TROP2 is normally expressed in placenta and foetal tissues but may also be detected in the normal stratified squamous skin epithelium, uterine cervix, oesophagus, and tonsillar crypts. TROP2 over expression is linked to aggressive tumour and increased mortality of cancer [123]. TROP2 is a single-pass type I membrane protein, containing a thyroglobulin type-1 repeat domain, potentially capable of binding IGF-II [124,125], and a putative EGF-like domain in its extracellular N terminus, the latter is presumably responsible for binding IGF-I, making TROP2 capable of inhibiting IGF-1R signalling (by sequestering the growth factors) [123,126] (Figure 6).

TROP2 is overexpressed in many cancers, both TROP2 gain-of-function and loss-of-function mutations are linked to tumour growth, presumably by promoting cell proliferation. Overexpression of TROP2 promotes tumour growth, but the lack of TROP2 following gene knock-out experiments results in the increased risk tumour development [127].

Sacituzumab govitecan, available under the brand name Trodelvy, is a humanized IgG1 monoclonal antibody against the TROP2 epithelial antigen, which is conjugated to topoisomerase inhibitor SN-38 through a cleavable linker. The binding of Sacituzumab govitecan to its target antigen on the TROP2-expressing cells results in the internalization of the antigen and the antibody into the cells, the release of topoisomerase I inhibitor and subsequent apoptosis. Sacituzumab govitecan is used for the treatment of metastatic triple-negative breast cancer and metastatic urothelial cancers [128]. Patients with low uridine diphosphate glucuronosyltransferase 1A1 activity have higher risk of developing adverse reactions including infusion-related reaction and therefore these patients require curious and pre-medications. At the time or writing this review, whilst this drug is available in the UK, due to funding issues it is currently only provided by NHS Scotland. However, the funding and approval of drugs is a dynamic process and their availability changes; for the up-to-date information the reader is directed to NICE.

### 6.2. Myeloid Cell Surface Antigen CD33

Myeloid cell surface antigen CD33 is a single-pass type I transmembrane protein receptor expressed on malignant bast cells in the majority of acute myeloid leukaemia cases. CD33 is a sialic-acid-binding immunoglobulin-like lectin, also known as Siglec-3 or gp67. The two extracellular Ig-like domains bind alpha-2,3- and alpha-2,6-linked sialylated glycoproteins and are involved in regulation of cell–cell interactions. The two cytoplasmic ITIM motifs are involved in negative regulation of the immune response [129]. CD33 is involved in maintaining immune cells in a resting state, including the repressive effect on monocyte activation. The specificity of CD33 for myeloid neoplasms makes it a good molecular target for monoclonal antibody therapy. Gemtuzumab ozogamicin is a humanized immunoglobulin IgG4 against CD33 protein antigen, that is covalently linked to a cytotoxic antibiotic N-acetyl gamma calicheamicin. Once bound to the CD33, the conjugated antibody undergoes internalisation, followed by the release of the cytotoxic chemotherapeutic agent that induces irreparable DNA damage and subsequently cell death [130]. Currently, in the UK, it is licenced in the treatment of CD33-positive acute myeloid leukaemia for people aged 15 and over [131].

## 7. Antibody Therapy Targeting Tumour-Specific Disialoganglioside

GD2 is a complex acidic glycolipid, that is usually expressed on the outer surface of cell membrane. GD2 molecules are formed of five monosaccharides linked to a sphingolipid ceramide. In normal tissues GD2 is expressed exclusively in the cerebellum in brain, in C nerve fibres in the peripheral tissues, and cutaneous melanocytes [132]. In addition to its normal structural function, these membrane glycolipid molecules may participate in cell growth, proliferation, and apoptosis. In abnormal tissues GD2 are overexpressed in tumours of neuroectodermal origin and ectodermal caners such as neuroblastoma, melanoma, glioma, small-cell lung cancer and soft tissue sarcoma, breast adenocarcinoma and phyllodes cancer [133]. Therefore, GD2 glycolipid is a good candidate for targeted cancer therapy. Dinutuximab (Qarziba, Unituxin) is an unconjugated mouse-human chimeric monoclonal IgG1 immunoglobulin against disialoganglioside GD2 that is used in the treatment of the neuroblastoma in patients aged one year and older. Dinutuximab targets the monosaccharides moiety of GD2 and induces cell-mediated and complement-mediated immune response towards the targeted tumour cells. Dinutuximab has a unique side effect profile that includes neuropathic pain, electrolytes, pyrexia, and myelosuppression [134].

## 8. Conclusions

Therapeutic antibodies are becoming of greater importance in the treatment of malignancies, especially those that show resistance to more traditional therapy such as chemotherapy agents and radiotherapy. More and more, these antibodies are showing that they can increase overall survival, disease-free survival and delay disease progression in several malignancies. More specifically, these antibodies also provide treatment options in non-resectable cancers and aid in delaying disease progression. Alongside this, many new therapeutic antibodies started being used as first-line treatment in many haematological malignancies. As we continue to understand the complex pathways that lead to carcinogenesis and the anti-immune properties that many cancers possess, this will continue to lead to more targeted therapeutic antibody treatments and potentially improved outcomes for patients with cancer.
